# Organic Nitrates: Past, Present and Future

**DOI:** 10.3390/molecules190915314

**Published:** 2014-09-24

**Authors:** Maria S. França-Silva, Camille M. Balarini, Josiane C. Cruz, Barkat A. Khan, Pabulo H. Rampelotto, Valdir A. Braga

**Affiliations:** 1 Biotechnology Center, Federal University of Paraíba, João Pessoa, PB 58037-760, Brazil; 2 Health Sciences Center, Federal University of Paraíba, João Pessoa, PB 58037-760, Brazil; 3 Faculty of Pharmacy and Alternative Medicine, The Islamia University of Bahawalpur, Bahawalpur 63100, Pakistan; 4 Interdisciplinary Center for Biotechnology Research, Federal University of Pampa, Antônio Trilha Avenue, P.O. Box 1847, São Gabriel, RS 97300-000, Brazil

**Keywords:** organics nitrates, nitrate tolerance, 1,3-dibutoxy-2-propyl nitrate

## Abstract

Nitric oxide (NO) is one of the most important vasodilator molecules produced by the endothelium. It has already been established that NO/cGMP signaling pathway deficiencies are involved in the pathophysiological mechanisms of many cardiovascular diseases. In this context, the development of NO-releasing drugs for therapeutic use appears to be an effective alternative to replace the deficient endogenous NO and mimic the role of this molecule in the body. Organic nitrates represent the oldest class of NO donors that have been clinically used. Considering that tolerance can occur when these drugs are applied chronically, the search for new compounds of this class with lower tolerance potential is increasing. Here, we briefly discuss the mechanisms involved in nitrate tolerance and highlight some achievements from our group in the development of new organic nitrates and their preclinical application in cardiovascular disorders.

## 1. Introduction

Nitric oxide (NO) is a volatile, multifunctional free radical with a short life span. It is synthesized by one of three isoforms of nitric oxide synthase and exerts its effect by the activation of the soluble glunylate cyclase (sGC), which culminates in the production of cyclic guanosine monophosphate (cGMP) and the activation of the cGMP-dependent kinase (PKG) [1,2]. It has been described that a reduction in NO bioavailability is involved in the pathophysiology of many cardiovascular diseases (CVD). In this context, the use of drugs capable of releasing NO is an effective approach while dealing with CVD [3].

Organic nitrates represent the oldest class of NO donors applied clinically. Among them, glyceryl trinitrate (GTN) is the main representative of the class, which also includes isosorbide dinitrate (ISDN), isosorbide 5-mononitrate (ISMN) and pentaerythritol tetranitrate (PETN) [4]. Despite the benefits of thesemolecules in treating CVD like *angina pectoris*, pulmonary hypertension and heart failure, their continued use may cause tolerance, which is, in fact, the main limitation to the use of organic nitrates [5,6]. Here we briefly discuss the past and the present perspectives in organic nitrates and highlight some preclinical tests performed by our group with a novel organic nitrate.

## 2. Historical Perspective: The Past

Glyceryl trinitrate, the first organic nitrate, was discovered in 1847 by Ascanio Sobrero. At that time, it was already described that headacheswere an unpleasant side effect of thissubstance [7]. In 1879, the English physician William Murrel described, for the first time, the beneficial effects of the GTN against *angina pectoris* [8]. Since then, GTN was established as a treatment for the relief of chest pain, although the exact mechanism of action of this compound remained obscure for about 100 years [9]. Murad and colleagues described, in 1977, that nitrates needed to release NO to present physiological effects [10]. Nitric oxide was only described as an endothelium-derived relaxing factor in middle 80s [11].

After to the introduction of GTN as a therapeutic agent for the treatment of angina pectoris, other nitro compounds with similar chemical properties have been developed. Most recent studies show that, in addition to angina, GTN and other organic nitrates such as ISDN and ISMN are able to improve left ventricular function in patients with congestive heart failure and pulmonary hypertension. Also, they show favorable effects on left ventricular remodeling after myocardial infarction and silent ischemia, in addition to reducing blood pressure alone or in combination with other drugs [12,13].

## 3. Mechanism of Action

Organicnitrates are potential NO donors in biological systems. In general, they do not exert their effect in cell-free systems and act as pro-drugs that need to be bioactivated by either enzymatic or non-enzymatic pathways to release NO. Enzymatic bioactivation of high-potency nitrates like GTN and PETN (tri- and tetranitrates, respectively) depends on the activity of cytosolic and/or mitochondrial aldehyde dehydrogenase (ALDH2), which converts them into nitrite and the denitrated metabolite. Non-enzymatic bioactivation of GTN involves its reaction with thiols from cysteine and cysteine derivatives or with ascorbate, which promote NO release. In contrast, low-potency nitrates such asISMN and ISDN (mono and dinitrates, respectively) undergo activation through a mechanism independent of ALDH2, which usually it occurs in endoplasmic reticulum via P450 enzymes [13,14,15].

Nitric oxide binds to soluble guanylate cyclase (sGC), leading to an increase in the intracellular concentration of cGMP, which in turn activates a specific cGMP-dependent protein kinase (PKG). Once PKG is active, it promotes the phosphorylation of diverse substrates like myosin light chain kinase (MLCK), sarco/endoplasmic reticulum Ca^2+^-ATPase (SERCA), plasma membrane Ca^2+^-ATPase and Na^+^/Ca^2+^ exchanger. All these events result in vasorelaxation and reduction in peripheral vascular resistance [16,17,18,19]. Several authors have presented evidence that the vascular relaxation mediated by NO can occur also by a mechanism independent of cGMP, due to direct activation of K^+^ channels [20].

## 4. Clinical Use of Organic Nitrates: The Present

### 4.1. Angina Pectoris

Angina can be defined as a chest pain or discomfort, usually attributed to myocardial ischemia. It is commonly associated with coronary heart disease and atherosclerosis, although it can also be related to cardiomyopathy or aortic stenosis. The severity of the discomfort does not necessarily relate to the severity of the underling coronary disease [21]. Angina is considered stable when it shows a regular pattern, being elicited by exertion or emotional stress and the pain is relieved by a few minutes of rest or after the use of nitrates.

Nitrates are the leading therapeutic class of drugs to treat *angina pectoris* [22]. They have been used even before the cardiovascular properties of NO have been established [7]. Organic nitrates exert their maximal vasodilator effects on venous capacitance vessels and large and medium coronary arteries, while small arterioles are less affected. The venodilation induced by these drugs increase the venous capacitance and reduce cardiac preload, which reduces left ventricular filling pressure and myocardium workload. The oxygen demand tomyocardium decreases and this is the key mechanism involved in the use of this class of drugs in angina [15]. At high doses, nitrates exert arterial vasodilator effects, leading to dilation in epicardial arteries. This promotes a redistribution of coronary blood flow from healthy regions to ischemic areas [23].

Short acting nitrates should be administrated to patients during acute *angina pectoris* symptoms or immediately before physical exercise to prevent angina. On the other hand, long lasting nitrates are recommended for the treatment of patients who remain symptomatic despite the use of aspirin, statins, beta receptor blockers or calcium antagonists [15].

### 4.2. Preeclampsia

Preeclampsia is a serious multisystem disorder characterized by new onset of hypertension, edema and proteinuria, typically after 20 weeks of pregnancy in otherwise normotensive woman. Although the cause of preeclampsia onset is not yet fully understood, endothelial dysfunction, defective NO synthesis, reduced NO bioavailability and reduced NO-mediated vasodilation play an important role in the pathophysiology of this condition [24,25,26]. Preeclampsia is associated with a substantive increase in morbidity and mortality for the mother and the baby and its complications usually include eclampsia, stroke, liver or kidney failure in the mother and poor growth and preterm birth for the baby [27].

Considering that disruption of NO/cGMP pathway is present in preeclampsia, the use of NO donors can be considered a potential pharmacological approach in this situation. In this context, organic nitrates have been used as a promising alternative to treat preeclampsia and without significant adverse cardiovascular effects. The disadvantage of these drugs relies in the possible tolerance development [25] and the efficiency of these drugs in preventing preeclampsia is controversial. Some studies show that they failed in prevent the establishment of preeclampsia, although other beneficial effects were observed, as the likehood of a complication-free pregnancy, decrease in resistance of blood flow to the placenta and improvement of uteroplacental blood flow [24,26].

The main adverse effect observed in the use of nitrates, especially GTN, is the increased risk of headache, which was sufficient severe to lead to the interruption of the treatment in some cases [27]. The successful use of GTN as a transdermal patch opens new therapeutic perspectives, once this approach has few side effects [26].

### 4.3. Pulmonary Hypertension

Pulmonary hypertension is defined as an increase in mean pulmonary arterial pressure (>25 mmHg) at rest [28]. Pulmonary hypertension is a severe and progressive disorder in which the pressure in the pulmonary arteries is increased due to sustained increase in pulmonary arterial resistance. It also culminates in progressive right ventricular dysfunction and death [7,29]. Although drugs aiming to increase vasodilation in pulmonary arteries have been used to treat pulmonary hypertension (for instance prostacyclin, endothelin receptor antagonists, phospodiesterase type 5 inhibitors) [29], organic nitrates have only been proposed as promising alternatives in experimental studies.

In this context, it has already been shown that organic nitrates are capable not only to exert direct vasodilator effects but also to inhibit the synthesis of pulmonary vasoconstrictors such as endothelin 1 and platelet-derived growth factor [30]. Brandler and colleagues successfully used a novel inhaled volatile organic nitrate in an experimental model [31]. They observed that the new compound was effective in promoting the relaxation of constricted pulmonary arteries and in alleviation of pulmonary hypertension. Further studies are necessary to establish the optimal dosing and long-term effects, however, this molecule holds the potential to be an alternative to inhaled NO, which presents toxicity issues and potential drawbacks [31].

## 5. New Perspectives: The Future

Nitrate tolerance is characterized by a reduction in the vasodilator effect of NO donors and the requirement of higher doses. Experimental approaches revealed that organic nitrates such as GTN, ISMN and ISDN induce tolerance. This phenomenon is not yet completely understood and may have several causes such as impaired bioactivation of the drug, desensitization of sGC/cGMP pathway and increase in reactive oxygen species (ROS), which inactivate both endogenous NO and NO released from nitrovasodilators. Furthermore, the process of tolerance is associated with the appearance of unfavorable cardiovascular changes such as increase in sympathetic activity and endothelial dysfunction. Apparently, the only organic nitrate in clinical used which does not induce tolerance or endothelial dysfunction is PETN. In fact, the antioxidant properties of this drug may be responsible, at least in part, for this advantageous characteristic [13,32,33,34].

**Figure 1:**
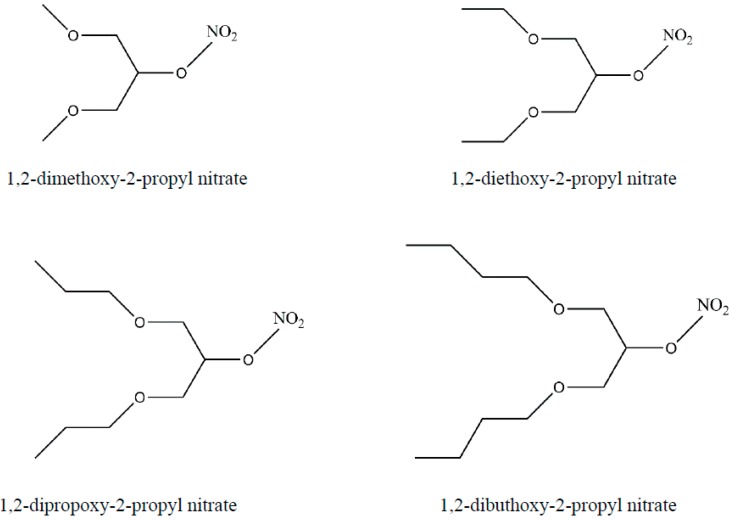
Structural formula of the new organic nitrates obtained from glycerin [35].

Considering that tolerance is the major limiting factor to clinical use of this class of drugs and that there is only one organic nitrate clinically available that does not induce tolerance, the search of new compounds unable to induce this undesirable effect has been increasing. Our group has recently evaluated new organic nitrates obtained from glycerin, which molecular structures can be observed in Figure 1.

**Figure 2:**
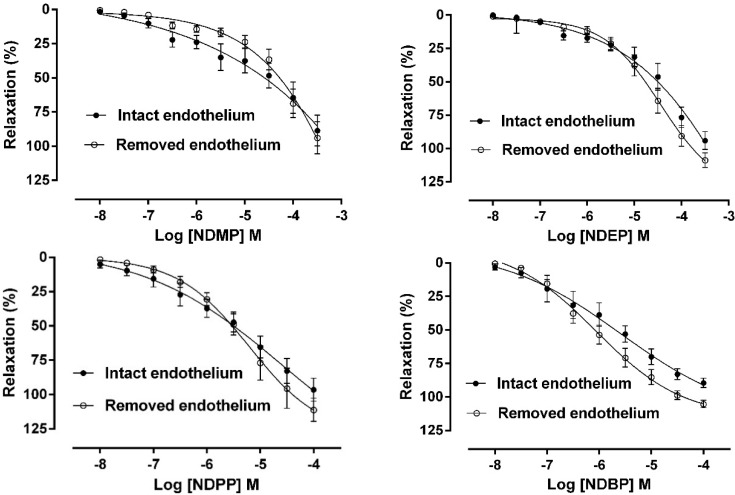
Concentration-response curves to new organic nitrates synthetized by our group (10^−8^–10^−4^ M or 3 × 10^−4^ M) in rat mesenteric artery rings (n = 6 per group). The vasorelaxant effect is expressed as a percentage of relaxation of phenylephrine-induced contraction [35].

Our first goal was to evaluate if these molecules could induce vascular relaxation, the main effect of organic nitrates. For this purpose, we performed concentration-response curves of the new NO donors in resistance rings of superior mesenteric artery from rats. We observed that all four compounds were able to induce vasorelaxation in a dose-dependent manner both in the presence or absence of functional endothelium, as shown in Table 1 and Figure 2.

**Table 1:** Maximum effect (ME) and sensibility (pD_2_) values regarding the vasorelaxant effect of the new organic nitrates derived from glycerin in superior mesenteric artery isolated from rats precontracted with phenyleprine, in the presence or absence of functional endothelium (n = 6 for each group) [35].

Compound	Intact Endothelium		Denuded Endothelium	
	(%) ME ± SEM	pD_2_ ± SEM	(%) ME ± SEM	pD_2_ ± SEM
NDMP	88.5 ± 11.2	4.7 ± 0.13	93.8 ± 11.7	4.4 ± 0.07
NDEP	94.1 ± 6.7	4.6 ± 0.08	108.8 ± 5.4	4.8 ± 0.06
NDPP	96.4 ± 8.3	5.5 ± 0.10	111.1 ± 8.5	5.4 ± 0.08
NDBP	89.5 ± 3.4	5.8 ± 0.10	105.4 ± 2.7	5.9 ± 0.06

**Scheme 1:**
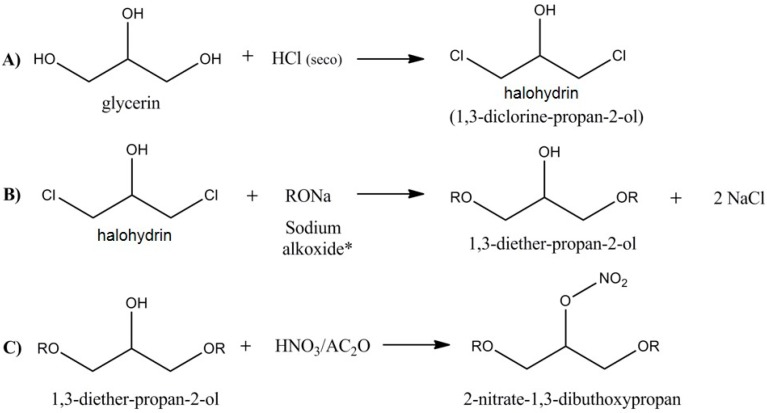
Synthesis of 1,3-dibutoxy-2-propyl nitrate [36].

Apparently, the vasodilator response tended to increase as the length of the organic chain added to glycerin increased. Because of that, we have focused our work on 1,3-dibutoxy-2-propyl nitrate (NDBP), which has a molecular formula of C_11_H_23_NO_5_ and a molecular weight of 249.3. Scheme 1 shows the steps of NDBP synthesis.

**Figure 3:**
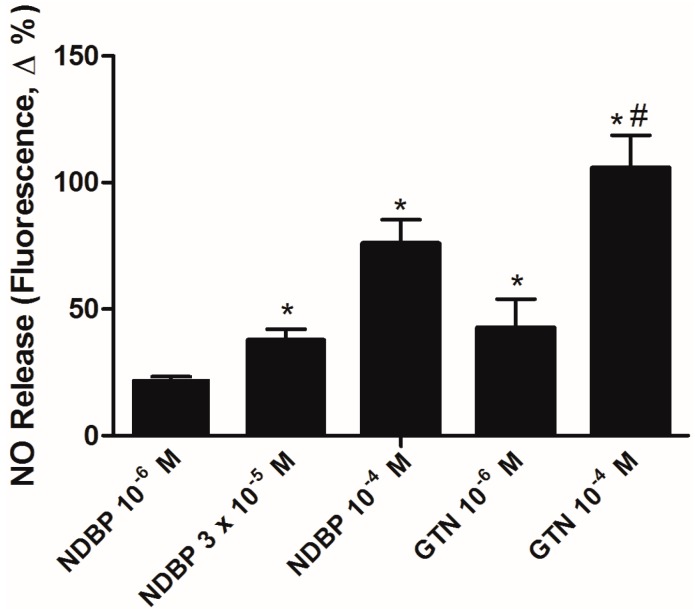
Nitric oxide generation by NDPB in vascular smooth muscle cells. * *p* < 0.05 *versus* basal fluorescence; # *versus* NDBP (10^−6^ M) and NDBP (3 × 10^−5^ M). Values are shown as mean ± S.E.M.

Using an *in vitro* pharmacological approach based on simultaneously use of NDBP and blockage of diverse steps of NO/sGC/cGMP pathway, we identified that vasorelaxation induced by NDBP is dependent of NO release, activation of sGC, generation of cGMP and activation of potassium channels [34], which is in accordance to what is expected from a NO-releasing drug. It was observed that NDBP is capable of increasing NO bioavailability in cultured vascular smooth muscle cells like other NO donors, such as GTN (Figure 3).

Considering that NDBP could be an alternative option to existing organic nitrates, our second goal was to analyze the effects of NDPB *in vivo*. Preclinical trials using five different doses of NDBP showed that the new compound presented hypotensive and bradicardic effects in a dose-dependent manner in normotensive and hypertensive non-anesthetized rats.These effects are of interest in a potential new NO donor, once the classical drugs of this class like GTN and sodium nitroprusside (SNP) can cause reflex tachycardia as a side effect. Parasympathetic blockage with atropine or vagotomy suggests that the reduction in blood pressure observed after NDPB administration depends not only to peripheral vasodilation but also to reduction in cardiac output due to increase in parasympathetic tone to the heart [36]. Further studies revealed that cardiovascular responses evoked by NDBP *in vivo* are dependent of NO release.When a NO-scavenger was used, both bradycardia and hypotension were attenuated [37].

Tolerance is one of the most important undesirable effects evoked by organic nitrates and is responsible for limiting the clinical use of this class of drugs. Thus, our third goal was to evaluate the ability of NDBP to induce tolerance, as previously described [35,38]. Preclinical *in vitro* approaches revealed that exposition of mesenteric artery rings to NDBP (10 µM or 100 µM) for 30 min prior to concentration-responses curves to this substance did not alter the vasorelaxant response, suggesting that NDBP did not induced tolerance [35].

Studies by our research group showed that intravenous treatment with NDBP (5 mg/kg) for three days did not affect vascular reactivity of superior mesenteric artery isolated from normotensive rats to cumulative addition of acetylcholine or SNP (unpublished data), suggesting that continuous exposure to NDBP, at least in the experimental conditions used, does not cause endothelial dysfunction or desensibilization of vascular smooth muscle to NO. From this data we can infer that treatment with NDBP for three days does not induce tolerance in the vascular preparation used, although more studies are needed to elucidate this phenomenon. One possible mechanism is that NDBP, like PETN, presents antioxidant properties.

## 6. Conclusions

Since deficiency in NO/sGC/cGMP pathway is involved in many pathological features of cardiovascular system, the use of NO-releasing drugs can figure as an option to treat these conditions. Nitric oxide donors are widely applied in clinical practice despite their undesirable effects, like tolerance. Recently, our group has developed a new organic nitrate with potential to be used clinically in cardiovascular disorders. Preclinical studies confirmed NDBP ability to release NO, leading to vasodilation. Besides, NDBP appears to also act in central nervous system to increase parasympathetic drive to the heart, culminating in reduced blood pressure. This new organic nitrate does not seem to induce tolerance neither endothelial dysfunction *in vitro*, suggesting that this molecule can figure as a new potential therapeutic approach for cardiovascular disorders. Further studies *in vivo* are required to investigate the effect of continuous treatment with NDBP on vascular oxidative stress in normotensive and hypertensive conditions and the ability of the compound to prevent pseudotolerance, other common effect of organic nitrates.
